# Embryo Genetics

**DOI:** 10.3390/genes12010118

**Published:** 2021-01-19

**Authors:** Carmen Rubio, Carlos Simón

**Affiliations:** 1 EmbryoGenetics Department, Igenomix, 46980 Valencia, Spain; 2 Professor of Obstetrics & Gynecology, Valencia University, 46010 Valencia, Spain; 3 Senior Lecturer PT, BIDMC Harvard University, Boston, MA 02215, USA; 4 Adjunct Clinical Professor, Department of Ob/Gyn, Baylor College of Medicine, Houston, TX 77030, USA

**Keywords:** embryo genetics, infertility, aneuploidies, monogenic disease, polygenic disease, blastocyst, endometrium, implantation

## Abstract

Advances in embryo and reproductive genetics have influenced clinical approaches to overcome infertility. Since the 1990s, many attempts have been made to decipher the genetic causes of infertility and to understand the role of chromosome aneuploidies in embryo potential. At the embryo stage, preimplantation genetic testing for chromosomal abnormalities and genetic disorders has offered many couples the opportunity to have healthy offspring. Recently, the application of new technologies has resulted in more comprehensive and accurate diagnoses of chromosomal abnormalities and genetic conditions to improve clinical outcome. In this Special Issue, we include a collection of reviews and original articles covering many aspects of embryo diagnosis, genome editing, and maternal–embryo cross-communication during the implantation process.

Infertility affects 15% of couples of reproductive age seeking to become parents, which accounted for 48 million infertile couples worldwide in 2010 [1]. The majority of these couples seek specialist medical care. In vitro fertilization (IVF) is the cornerstone of infertility treatment. European statistics indicate that approximately 500,000 IVF cycles are performed annually, resulting in the birth of 100,000 infants, or nearly 5% of all babies born in countries such as Denmark. 

Having a baby is just the first challenge to overcome in the reproductive journey; the next and most important is to give birth to a healthy baby free of preventable genetic conditions. Genetic disorders affect 1% of live births and are responsible for 20% of pediatric hospitalizations and 20% of infant mortality. Many such disorders are caused by recessive or X-linked genetic mutations carried by 85% of the human population. Because assisted reproduction has provided us with technologies such as IVF that provide access to human embryos, certain genetic diseases were initially screened by selecting sex. The first live births following preimplantation genetic testing (PGT) to identify sex for X-linked disorders were reported by Alan Handyside in 1990 [2]. This ground-breaking work identified male embryos and selectively transferred unaffected normal or carrier females to avoid genetic diseases, paving the way to extend the concept to PGT for monogenic diseases (PGT-M), including Mendelian single-gene defects (autosomal dominant/recessive and X-linked dominant/recessive), severe childhood lethality or early-onset disease, cancer predisposition, and Human Leukocyte Antigen (HLA) typing for histocompatible cord blood stem cell transplantation. 

Later, we moved on to identifying and selecting euploid embryos by analyzing all 23 pairs of chromosomes in 4–8 cells from the trophectoderm, known as PGT for aneuploidy (PGT-A). PGT-A currently leverages next-generation sequencing (NGS) technologies to uncover meiotic- and mitotic-origin aneuploidies affecting whole chromosomes, as well as duplications/deletions of small chromosome regions. A further step forward was the use of structural chromosome rearrangements (PGT-SR) to identify Robertsonian and reciprocal translocations, inversions, and balanced vs. unbalanced rearrangements. Another advancement came with PGT for polygenic risk scoring (PGT-P). This technique has taken us from learning how to read simple words to beginning to understand poetry (i.e., evolving from PGT-M/A/SR to PGT-P for multifactorial, polygenic risk prediction). Common multifactorial diseases, such as diabetes, coronary heart disease, and cancer, are caused by a combination of environmental, lifestyle, and genetic factors; risk scores are now being generated to predict the likelihood of such complex, later-life diseases in embryos. Moreover, we are moving from embryo selection to intervention because the human genetic code is not only readable and writable, but also hackable. Indeed, gene editing is now possible using tools such as (CRISPR)/CRISPR-associated (Cas9), which is applicable to all species, including human embryos. 

This Special Issue traverses the field of embryo genetics in ten papers: four reviews and six original articles addressing specific aspects of developments in technology, clinical application, and basic research.

The first review introduces the reproductive journey in the genomic era, from preconception to childhood, leveraging NGS as a genomic precision diagnostic tool to understand the mechanisms underlying genetic conditions, which account for 20–30% of all infant deaths and more than 50% of clinical miscarriages [3]. Genome-wide technologies are applied at different stages of the reproductive health lifecycle from preconception carrier screening and preimplantation genetic testing, to prenatal and postnatal testing. 

Six articles cover preimplantation genetic testing at the chromosome and gene levels. A review for genetic disorders covers the evolution of this technology as an established alternative to invasive prenatal diagnosis, as well as future innovations [4]. The development of new algorithms and the declining costs of sequencing are propelling PGT to a sequencing-based, all-in-one solution for PGT-M, PGT-SR, and PGT-A. Along this line, this Special Issue includes an original manuscript for combined PGT-M and PGT-A in autosomal dominant polycystic kidney disease (ADPKD). ADPKD can manifest extrarenally and as seminal cysts that have been associated with male infertility in some cases [5]. The results of this study indicate that AMA couples who are also ADPKD patients have an increased risk of generating aneuploid embryos, but ADPKD-linked male infertility does not promote an increased aneuploidy rate. In a third article, the possibility of testing embryos not only for monogenic diseases, but also for polygenic conditions (PGT-P) is presented, with a strategy of disease relative risk reduction to evaluate the potential clinical utility of embryo selection with PGT-P [6]. The results demonstrate the potential for simultaneous relative risk reduction for all diseases tested in parallel, which include diabetes, cancer, and heart disease, and indicate applicability beyond patients with a known family history of disease.

Of the three articles related to embryo chromosomal abnormalities, the first is a review of state-of-the-art methods for PGT-A, mosaicism, and PGT-SR that reinforces the idea that there is a high incidence of chromosomal abnormalities in early human embryos, resulting in low success rates with assisted reproductive technologies [7]. Chromosomal anomalies are also responsible for a large proportion of miscarriages and congenital disorders. The review covers efforts from 2000–2020 to improve technology to accurately identify embryos containing chromosomal abnormalities. The second article describes an optimized NGS approach for PGT-A and PGT-SR with special emphasis on mosaicism and the development of tailored algorithms and diagnostic tools to identify different levels of mosaicism objectively in order to avoid subjectivity in the diagnosis [8]. The third article addresses different extrinsic factors related to the IVF cycle that could affect the incidence of overall aneuploidy, whole uniform aneuploidy, mosaicism, and segmental aneuploidy. Female and male parental age, ovarian response, embryo vitrification, and sperm concentration were considered in a multivariate analysis [9]. 

Finally, the development of novel genome editing tools has unlocked new opportunities for gene editing at the embryo level. We incorporate a review of new developments in genome editing techniques to modify specific regions of the genome [10]. Among genome editing tools, the CRISPR/Cas system has proven to be the most popular for both basic research and clinical purposes and was the topic for the Nobel Laureate in Chemistry 2020.

To end this Special Issue, we highlight that other players beyond the embryo are crucial in the reproductive journey. The endometrium is particularly important, with implantation failure resulting from suboptimal endometrial receptivity. As pregnancy progresses, the uterus continues to communicate closely with the embryo/fetus. Recent progress in the availability of high-throughput techniques, including transcriptomics, proteomics, and metabolomics, has allowed the simultaneous examination of multiple molecular changes, enhancing our knowledge in this area. This review covers known mechanisms of mother–embryo cross-communication identified from animal and human studies [11]. Coverage of this topic concludes with an original research article describing the identification and characterization of extracellular vesicles and their DNA cargo that is secreted during embryo development in a murine model [12]. The authors conclude that murine blastocysts secrete extracellular vesicles containing genome-wide sequences of DNA to the medium, reinforcing the relevance of studying these vesicles and their cargo at the preimplantation stage, where secreted DNA may aid in the assessment of the embryo before implantation.

We can conclude that, in the coming years, genetics will dramatically change and improve the field of reproduction and infertility treatments by means of precision medicine.
